# A review of potassium channels in bipolar disorder

**DOI:** 10.3389/fgene.2013.00105

**Published:** 2013-06-11

**Authors:** Jennifer T. Judy, Peter P. Zandi

**Affiliations:** ^1^Department of Psychiatry, Johns Hopkins School of MedicineBaltimore, MD, USA; ^2^Department of Mental Health, Johns Hopkins Bloomberg School of Public Health, Johns Hopkins UniversityBaltimore, MD, USA

**Keywords:** potassium channels, bipolar disorder, channelopathy, ion channels, mood disorders

## Abstract

Although bipolar disorder (BP) is one of the most heritable psychiatric conditions, susceptibility genes for the disorder have yet to be conclusively identified. It is likely that variants in multiple genes across multiple pathways contribute to the genotype–phenotype relationship in the affected population. Recent evidence from genome-wide association studies implicates an entire class of genes related to the structure and regulation of ion channels, suggesting that the etiology of BP may arise from channelopathies. In this review, we examine the evidence for this hypothesis, with a focus on the potential role of voltage-gated potassium channels. We consider evidence from genetic and expression studies, and discuss the potential underlying biology. We consider animal models and treatment implications of the involvement of potassium ion channelopathy in BP. Finally, we explore intriguing parallels between BP and epilepsy, the signature channelopathy of the central nervous system.

## GENETIC EPIDEMIOLOGY

Bipolar disorder (BP) is a disabling psychiatric condition with a considerable public health impact, affecting 1–2% of the general population (Weissman et al., 1996). Family, twin, and adoption studies show that genetic factors play a leading role in is etiology. Family studies suggest that compared to the general population the risk of BP is 5–10 times greater in siblings of a proband with the disorder (Craddock and Jones, 1999) and twin studies provide estimates of heritability for BP that range from 80 to 90%, ranking it among the most heritable of psychiatric disorders (Barnett and Smoller, 2009). However, conclusive findings from molecular studies to identify relevant genetic factors have proven elusive, and the genetic architecture remains unresolved. Although results from sequencing analyses may reveal rare variants with large effects on the level of the individual, single variants are not likely to influence BP risk at the population level (Craddock and Sklar, 2009). Rather, multiple genetic phenomena likely contribute to BP risk, including the additive and interactive effects of many variants, each contributing a small effect, as well as heterogeneity across populations. This has lead to an interest in identifying the molecular pathways that are disrupted by these risk variants, any number of which may underlie an individual’s susceptibility to BP (Askland et al., 2009).

## GENOME-WIDE ASSOCIATION STUDIES

Encouraged by the evidence from genetic epidemiology studies, considerable effort has been expended over the past two decades to identify susceptibility genes for BP. The completion of the Human Genome Project and advances in high-throughput genotyping technology spurred a new generation of genome-wide association studies (GWAS) making it possible to test for genetic associations at sites of common polymorphic variation in an unbiased manner across the entire genome. The first GWAS of BP with individual level genotype data came from the Wellcome Trust Case Control Consortium (WTCCC; Wellcome Trust Case Control Consortium, 2007). The WTCCC simultaneously conducted a GWAS of seven different disorders, including BP. Two thousand cases for each disorder and a common set of 3,000 controls were genotyped at <500,000 single-nucleotide polymorphisms (SNPs) across the genome. The analysis of data on BP revealed no findings that were considered genome-wide significant. Soon afterward, another GWAS of BP was reported from the Systematic Treatment Enhancement Program for Bipolar Disorder (STEP-BD; Sklar et al., 2008). In this study, a total of 1,461 cases with BP from the STEP-BD trial and the University College of London (UCL) and 2,008 controls were genotyped. As with the WTCCC, none of the findings were genome-wide significant. Given the heritability of BP, the lack of genome-wide significant findings and generally inconsistent results from these early GWAS highlighted the fact that the common variants are likely to represent genetic effects of modest size.

It was not until large samples from several different studies were combined in a mega-analysis that compelling findings for BP began to emerge. In 2008, Ferreira et al. (2008) reported a GWAS of 4,387 BP cases and 6,209 controls from the WTCCC, STEP-UCL, and ED-DUB-STEP2 datasets with over 1.8 million genotyped and imputed SNPs. This was the largest GWAS to date and it reported the first genome-wide significant association in BP [rs10994336, odds ratio (OR) = 1.45, *p* = 9.1 × 10^-9^] at *ANK3* (ankyrin G). Schulze et al. (2009) subsequently replicated the findings with *ANK3* in a candidate gene study using a sample that had previously been part of a pooled GWAS (Baum et al., 2008). They found significant associations with two SNPs in *ANK3*, including the SNP reported by Ferreira et al. (2008; rs9804190, OR = 1.32, *p* = 3 × 10^-6^; rs10994336, OR = 1.54, *p* = 1.7 × 10^-5^). The two SNPs were ~340 kb apart with little linkage disequilibrium between them, suggesting the possibility of independent loci contributing to allelic heterogeneity.

Three GWAS (Scott et al., 2009; Smith et al., 2009; Lee et al., 2010) were subsequently reported, including one (Smith et al., 2009) of 1,001 BP cases and 1,033 controls of European-American ancestry and 345 BP cases and 670 controls of African-American ancestry from the National Institute of Mental Health (NIMH) Genetic Association Information Network (GAIN), a second (Scott et al., 2009) on 2,076 BP cases and 1,676 controls of European-American ancestry that partially overlapped with the first, and a third (Lee et al., 2010) on 1,000 BP cases and 1,000 controls of Han Chinese ancestry. None of the findings from these three GWAS met criteria for genome-wide significance, but provided further confirmatory evidence for an association with *ANK3*. Scott et al. (2009) carried out a fixed effects meta-analysis of rs10994336 with the previously published findings and provided the strongest evidence yet of association with *ANK3* (OR = 1.47, *p* = 1.1 × 10^-10^).

To improve the power to detect susceptibility variants in BP and other complex psychiatric disorders, a landmark initiative referred to as the Psychiatric GWAS Consortium (PGC) was undertaken to assemble existing samples of BP, schizophrenia, depression, attention-deficit hyperactivity disorder (ADHD), and autism and carry out the largest GWAS ever conducted with these disorders (Psychiatric GWAS Consortium Coordinating Committee et al., 2009). The mega-analysis led by the PGC-BP (Psychiatric GWAS Consortium Bipolar Disorder Working Group, 2011) brought together an international sample of 7,481 BP cases and 9,250 controls with imputed genotype data on ~2.5 million SNPs. The top finding was again in *ANK3* with an OR = 1.35 (*p* = 7.1 × 10^-9^) at rs10994397, which is approximately 99 kb from rs10994336, the SNP that was highlighted in the original Ferreira et al.’s (2008) study. When combined with an independent replication sample of 4,496 BP cases and 42,422 controls, the most significant finding to emerge was at rs4765913 in *CACNA1C* with an OR = 1.14 (*p* = 1.52 × 10^-8^).

The convergence of findings around *ANK3* and *CACNA1C* has suggested a biologically plausible etiologic mechanism in BP. *ANK3* is an adaptor protein that regulates the distribution of voltage-gated ion channels, and *CACNA1C* is the alpha 1C subunit of the L-type voltage-gated calcium channel. This lent further support to the burgeoning hypothesis that BP may be an ion channelopathy (el-Mallakh and Wyatt, 1995; El-Mallakh and Huff, 2001; Askland, 2006; Askland and Parsons, 2006; Casamassima et al., 2010). Pathway-based analyses of GWAS data, which collectively tests for associations of SNPs across sets of genes within defined molecular pathways instead of testing each SNP independently (Askland et al., 2009), has provided further evidence for this hypothesis.

A recent pathway-based analysis (Askland et al., 2009) was carried out with data from the NIMH GAIN and WTCCC GWAS using gene sets based on gene ontology (GO) categories. Results from this analysis implicated genes involved in the structure and regulation of ion channels. Of the 16 gene sets that were statistically significant (*p* < 0.01) in the NIMH GAIN discovery sample, nine were ion channel/binding/transporter sets. Eight of these nine were replicated in the WTCCC sample. Notably, the most significant group in the NIMH GAIN sample, which was also replicated in the WTCCC sample, was a gene set involved in voltage-gated ion channel activity. Since only four genes overlap in the gene sets between samples, it appears that the shared molecular function (mediation of voltage-gated ion channel activity) rather than a few highly significant results common across studies drove the results.

Another pathway-based analysis (Le-Niculescu et al., 2009) used a convergent functional genomics (CFG) approach to identify gene sets of interest. They first extracted nominally significant signals (*p* <0.05) from three GWAS studies (WTCCC, German, and NIMH GAIN datasets; Fangerau et al., 2004; Wellcome Trust Case Control Consortium, 2007; Baum et al., 2008), and prioritized potential candidates based on independent, converging lines of evidence from various bioinformatics resources. Results implicated gene sets involved in calcium and potassium regulation, as well as cellular adhesion, thus providing further evidence implicating dysregulation of ion channel machinery in BP.

In the largest GWAS of psychiatric illness to date, a recent analysis from the PGC Cross-Disorder Group (Cross-Disorder Group of the Psychiatric Genomics Consortium et al., 2013) combined various approaches to examine a potentially shared genetic contribution to five psychiatric disorders: major depressive disorder, bipolar disorder, schizophrenia, autism spectrum disorders, and ADHD (total *N* = 33,332 cases and 27,888 controls). Methods included multinomial logistic regression with model selection, polygenic risk score analysis, pathways analysis, and an enrichment analysis of expression quantitative trait loci (eQTL) data. In the primary analysis, two of the four genome-wide significant results were in calcium channel genes (*CACNA1C* and *CACNB2*), and model selection supported a contribution of these variants to multiple disorders. Additionally, results from the pathways analysis suggested that genes involved in calcium channel activity have pleiotropic effects among multiple disorders. These findings suggest that variants which alter calcium channel functioning contribute not only to BP, but also to a shared, fundamental mechanism that contributes to a broader definition of psychopathology.

## GENETIC FINDINGS WITH POTASSIUM ION CHANNELS

Because of the findings with *CACNA1C*, most of the attention has understandably focused on the role of calcium ion channels in BP. However, the possibility that potassium ion channels may also contribute to the etiology of BP was suggested by a re-analysis of GWAS data from the NIMH GAIN sample (reported in a companion paper in this issue). Given that *ANK3* encodes a protein known to molecularly interact with other membrane bound proteins in neurons, we sought to test whether interactions between *ANK3* and genes encoding these other proteins may contribute to BP susceptibility. Using the STRING 9.0 bioinformatics database (Szklarczyk et al., 2011), which collates existing evidence of protein–protein interactions, we identified putative interactors with ANK3. We then tested for interactions between SNPs in *ANK3* and in these interacting proteins in association with BP. The most significant findings were between *ANK3* and *KCNQ2* (*p* = 3.18 × 10^-8^), which remained significant after accounting for multiple testing. These interactions were driven by two SNPs in *KCNQ2*, rs2282150 (an intronic SNP) and rs2297385 (a synonymous coding SNP), interacting with 16 different SNPs in *ANK3* that all fall within the boundaries of ankyrin repeats, which are functional domains known to mediate protein–protein interactions. We were able to replicate these associations using data from the WTCCC sample. We also found suggestive evidence of interactions between *ANK3* and *KCNQ3* (*p* = 1.86 × 10^-5^). *KCNQ2* and *KCNQ3* encode proteins that form hetero-tetramer complexes making up the voltage-gated potassium ion channels in neurons.

Interestingly, previous genetic studies have implicated potassium related genes in BP. For example, of the over 40 linkage regions that had been reported in genome-wide linkage studies of BP (Newton, 2007), one of the most prominent implicated chromosome 8q24, a region that includes *KCNQ3*. The 8q24 region initially reached a logarithm (base 10) of odds (LOD) score of 2.39 in a genome-wide scan by the Johns Hopkins Mood Disorders Research Group (Friddle et al., 2000). This finding was strengthened by including additional pedigrees (McInnis et al., 2003) and markers [Avramopoulos et al., 2004; non-parametric linkage (NPL) = 3.13 and 3.25, respectively]. It was also replicated in an independent sample by a German group (Cichon et al., 2001; LOD = 3.6). A meta-analysis using primary genotype data from 11 genome-wide linkage scans again indicated 8q as one of only two significant regions in the genome (LOD = 3.4; McQueen et al., 2005). Zandi et al. (2007) fine mapped a 3.4-Mb region under the linkage peak with 249 informative SNPs. This family-based association analysis with 155 nuclear families identified a nominally significant SNP (rs1901090, *p* < 0.05) and haplotype (rs2945733–rs1901090, *p* = 0.003) near the gene. Finally, this region was further probed with 2,756 observed and 15,552 imputed genotypes (Zhang et al., 2010), the most significant of which (rs2673582, *p* = 4.80 × 10^-5^) lies 27 kb upstream of *KCNQ3*.

*KCNQ2* has also been implicated in BP by candidate gene association studies. One study (Borsotto et al., 2007) found two significant associations with SNPs in KCNQ2 (*p* < 0.05). The results from this study are particularly interesting given the functional consequences of these variants within *KCNQ2*, which suppressed the potassium channel activity. The authors hypothesized that this might result in hyperexcitable neurons, which are characteristic of manic and hypomanic states.

Other candidate gene studies of BP have implicated *KCNN3*, a voltage-independent calcium-activated potassium channel, which is thought to mediate neuronal excitability via the slow component of synaptic afterhyperpolarization that follows an action potential event. The highly polymorphic CAG-repeat array in exon 1 had been implicated in BP (Chandy et al., 1998; Wittekindt et al., 1998; Hawi et al., 1999; McInnis et al., 1999; Rohrmeier et al., 1999). However, a meta-analysis of pooled evidence from these individual studies was null (Glatt et al., 2003). The authors of the meta-analysis noted that the polymorphism in question, or one in LD with it, might yet be associated with a subphenotype of the disorder, such as anticipation or symptom severity. Indeed, a more recent analysis of a schizophrenic sample found an association between the long CAG repeats (which reduce the channel’s functioning) and better cognitive performance on tasks that measured the ability to discriminate, select, and execute (Grube et al., 2011).

A series of gene expression studies have provided further evidence for a role of potassium ion channels in BP. Using post mortem striatum samples of human brains from the Stanley Foundation Brain Collection [including the nucleus accumbens (Str-NAc) and the lateral cerebellar hemisphere], one group (Smolin et al., 2012) analyzed expression of 72 different ion channel subunits. They found altered gene expression patterns in BP, with a significant up-regulation of *KCNQ2* (fold change: 1.51, *p* = 0.039677), *KCNQ3* (fold change: 7.00, *p* = 0.005305), *KCNA4* (fold change: 2.03, *p* = 0.002), as well as significant up-regulation of two α- and β-subunits of voltage-gated type I sodium channel genes (*SCN1A* and *SCN1B*) in the lateral cerebellar hemisphere. In the Str-NAc, they found a significant down-regulation of *KCNS3* (fold change: -1.67, *p* = 0.00082), *KCNA1* (fold change: -1.59, *p* = 0.00396), and up-regulation of *KCNN3* (fold change: 1.57, *p* = 0.04719). Due to the known ability of mood-stabilizing drugs to directly interfere with ion channel activity, they also looked for differential expression among the 72 genes between subjects on versus off mood stabilizers. Interestingly, the only significant genes were *KCNQ3* (*p* = 0.012) and *SCN1B* (*p* = 0.004) in the cerebellum.

In a gene expression study using an animal model, whole-brain mRNA from mice treated with antipsychotics (clozapine, haloperidol, or olanzapine) was examined (Duncan et al., 2008). The results of the study suggested that treatment with antipsychotics altered regulation of two genes encoding subunits of the K_v_1 potassium voltage-gated channel, shaker related subfamily: *KCNA1* and *KCNAB1*, as well as the K_v_ channel-interacting protein *KCHIP*. The authors of the study concluded that the modulation of neuronal voltage-gated ion channels, particularly potassium and sodium, contribute to the mechanism of action for antipsychotics via their effects on neuronal electrical activity and neurotransmission.

In two other gene expression studies with animal models, the effect of electroconvulsive therapy (ECT) on the expression levels of voltage-gated potassium channel subunits in rat brain was examined (Pei et al., 1997; Hjaeresen et al., 2008). ECT is a pro-convulsive treatment in which electrical currents are passed through the brain in order to induce a brief seizure (Mayo Clinic, 2010). However, over time, ECT actually has anticonvulsive properties (Sackeim, 1999). Its primary indication is for the treatment of major depression, although there is anecdotal evidence for its use in treating mania (Versiani et al., 2010). The first study found that the expression levels of the potassium channel genes were affected by both chronic and acute ECT, and that these effects were specific to channel type, brain region, and timing (Pei et al., 1997). The second study also found that chronic ECT significantly (*p* < 0.001) increased expression levels, again in a channel subtype and brain region specific manner (Hjaeresen et al., 2008). Since potassium channel genes are integral to the regulation of membrane potentials, the authors concluded that the increased levels of mRNA might be at least partially relevant to the seizure threshold elevating effect of ECT.

## ION CHANNELS AS THERAPEUTIC TARGETS

Ion channels provide an attractive target for pharmacological intervention, given their diverse and ubiquitous roles in a broad range of physiological processes. Among all drugs with known targets, approximately 13.4% have their primary therapeutic action at ion channels. This ranks ion channels second as a target class, behind G protein-coupled receptors (GPCRs; Overington et al., 2006).

Accumulating evidence suggests that existing treatments for BP may potentially exert their therapeutic action via ion channel regulation (Gould et al., 2004). Lithium, a simple monovalent cation, is a first-line treatment for BP (Quiroz et al., 2004). Although its mechanisms of action are not entirely clear (O’Brien and Klein, 2009), there is some evidence that it affects ion channel functioning. A recent investigation of *in vitro* lithium treatment in mouse brain tissue determined that, at therapeutic concentrations, lithium entered the cell through sodium channels and suppressed the outward membrane current, which directly affected membrane excitability (Butler-Munro et al., 2010). The authors concluded that ion channels that regulate neuronal excitability may be a common target among BP treatments. Lithium is also a direct and potent inhibitor of glycogen synthase kinase 3β (GSK3β; Klein and Melton, 1996; Quiroz et al., 2004). GSK3β has many functions, one of which is to phosphorylate the voltage-gated potassium channel KCNQ2. It has been shown *in vitro* that phosphorylation of KCNQ2 decreases the activity of the channel and subsequently reduces the M-channel current (Borsotto et al., 2007). It is intriguing to speculate that lithium may have therapeutic effect by blocking this downstream phosphorylation of KCNQ2. Antiepileptic drugs (AEDs) such as valproate, lamotrigine, and carbamazepine have a long history of successfully treating BP, and may similarly affect the regulation of neurotransmission and action potential firing via mediation of ion channel functioning. The AEDs that are effective against BP likely have multiple molecular targets with variable contributions to the drugs’ efficacy (Perucca, 2005). However, it is notable that they are all known to diminish the flow and accumulation of sodium ions in the cell (El-Mallakh and Huff, 2001).

Voltage-gated potassium channels, specifically the Kv7 channels, are particularly attractive targets for novel therapeutics for BP. In addition to their functionally relevant roles within neurons, they offer the ability for selective intervention (Brown and Passmore, 2009). For example, ezogabine (EZG) is a neuronal potassium channel enhancer that was recently approved by the FDA (Food and Drug Administration) to treat partial epilepsy (Little, 2009). It has been shown (Wickenden et al., 2000) that EZG activates the KCNQ2/KCNQ3 hetero-tetramer ion channel complex, which in turn promotes the M-current and thereby stabilizes action potential firing. This action likely accounts for the drug’s anticonvulsant properties (Borsotto et al., 2007).

Several groups have studied the potential mood-stabilizing effects of EZG and other Kv7 channel enhancers in a series of animal studies. One study (Redrobe and Nielsen, 2009) used a mouse model of mania and determined that EZG (a non-selective opener) and ICA-27243 (a selective Kv7.2/3 opener), but not BMS-204352 (a selective Kv7.4–Kv7.5 and Kv7.7/3 heteromer opener) were able to reduce motility in the hyperactive mice, a paradigm for antimanic properties in drugs. In another study employing the D-amphetamine and chlordiazepoxide (AMPH + CDP) mouse model (Kristensen et al., 2012), EZG reduced cerebral glucose metabolic activity while increasing phosphor-serine-9 levels of GSK3β with a brain regional signature that mirrored lithium and valproate.

Ezogabine has also been evaluated for the acute treatment for mania in a small (*N* = 10) open label pilot study of treatment resistant in patients with BP type I (Amann et al., 2006). Despite the limited sample, the brevity of the study design, and the severity of illness, improvement in mania scores was observed in four patients. The treatment was well tolerated with no depressogenic effects, indicating that further study may be warranted.

## NEURONAL CHANNELOPATHIES

The accumulating evidence from molecular genetic studies provides a plausible case that BP may be due, at least in part, to a channelopathy. The term “channelopathy” is a relative newcomer to the medical lexicon, referring to disorders that result from defective ion channel functioning (Ashcroft, 2000). Ion channels are complex, membrane-spanning glycoproteins composed of separate pore-forming and accessory subunits (Ashcroft, 2006). These pores allow the selective, passive transport of charged ions through the hydrophobic cell membrane, which is otherwise relatively impermeable to ions. While the actual movement of the ions themselves is largely inconsequential, it is the resulting transmembrane current that elicits the electrical excitability that defines their physiological contributions (Gargus, 2003). These currents allow ion channels to orchestrate electrical signals such as action potentials in neurons (El-Mallakh and Huff, 2001). Action potentials, which result from transient changes in the membrane’s permeability to sodium and potassium ions, are electrical events that encode and transmit information, facilitating neuronal communication via neurotransmitters (Stuart et al., 1997). Sodium (Na^+^) channels are responsible for the initial depolarization (or rising) phase of the action potential, and potassium (K^+^) channels are responsible for the repolarizing phase, which returns the membrane to its resting potential (Barnett and Larkman, 2007).

Ion channel genes are quite prominent, representing 1–2% of the entire genome (Clare, 2010; Stelzer et al., 2011), and most display tissue-specific expression. Ion channels are extraordinarily diverse, suggesting a high degree of functional specificity (Rudy, 1999). Their diversity is often amplified by the various combinations of heteromers between family members that co-assemble to form functional units of the channels. Additionally, variations in their different constituent subunits allow for modification of their functioning, through alternative splicing (Gargus, 2003). Therefore, while ion channels play a role in a broad range of physiological processes in various tissues, genetic variation in the genes that code for them often result in tissue-specific diseases (Cannon, 2006).

Potassium channels in particular figure prominently in human channelopathies (Ryan and Ptacek, 2010). Their structural and functional diversity, which is greater than any other group of ion channels (Kress and Mennerick, 2009), underscores the variety of physiological function that they modulate (Lawson, 2000). In particular, variants within the KCNQ gene family (*KCNQ1–5*, also referred to as Kv7.1–5) have figured prominently in human channelopathies (Gargus, 2006). Of the ten potassium channel genes that have proven associations with human disease, four are members of the KCNQ subfamily (Borsotto et al., 2007). Additionally, four of the five KCNQ gene family members have been associated with different hereditary diseases. The diversity, ubiquity, and functional significance of the KCNQ gene family imply that they are likely to play a role in the etiology of more diseases than have already been identified (Sanguinetti and Spector, 1997; Borsotto et al., 2007).

As suggested above, two members of the KCNQ gene family (*KCNQ2* and *KCNQ3*) may be particularly relevant to BP etiology. *KCNQ2* and *KCNQ3* are both expressed in the brain and form hetero and homomeric voltage-gated potassium channels. ANK3 helps to direct the localization of the KCNQ2/KCNQ3 channels to the axonal initial segment (AIS) of neurons and nodes of Ranvier. Indeed, such localization is abolished in *ANK3* knock-out mice (Pan et al., 2006). The crucial role of KCNQ2 and KCNQ3 at the AIS is to form M-type channels that mediate sub-threshold M-currents. These M-currents stabilize the neuronal resting potential and inhibit repetitive firing of action potentials and thereby prevent neuronal hyperactivity (Delmas and Brown, 2005). Thus, disruption of normal KCNQ2/KCNQ3 activity may be salient to the etiology of BP. Supporting this hypothesis are animal model studies which have shown that the suppression of the M-current by dominant-negative mutations in *KCNQ2* lead to hyperexcitability of neurons, morphological changes of the hippocampus, a notable decline in cognitive function, and behavioral changes corresponding to hyperactivity (Peters et al., 2005). Interestingly, mutations for benign familial convulsions, a rare autosomal dominant inherited form of epilepsy, have been identified in *KCNQ2* (Singh et al., 1998).

## BIPOLAR DISORDER AND EPILEPSY

Bipolar disorder has many overlapping features with epilepsy (Amann and Grunze, 2005; Askland, 2006), which has been referred to as the “signature channelopathy phenotype in the CNS” (Gargus, 2006). Epilepsy is a brain disorder involving repeated and unprovoked seizures, which are periods of disturbed brain function caused by abnormally excited electrical signals (Vorvick and Jasmin, 2010). Epilepsy has a number of subtypes that are proven channelopathies (Heron et al., 2007), and as a result it can be used as a model to illustrate other central nervous system (CNS) conditions that may arise from disruptions of ion channel functioning (Gargus, 2003). In particular, there are many intriguing parallels between BP and epilepsy that suggest common neurobiological basis, specifically implicating ion channel functioning (Mazza et al., 2007).

One of the most striking similarities between BP and epilepsy involves the course of illness for both disorders, which involves a predominantly normal phenotype punctuated by sporadic interruption of rhythmic functioning (Gargus, 2006). The episodic course of both disorders often progresses to become chronic, and sometimes treatment resistant (Mula et al., 2010). The progression of these conditions known as “kindling,” whereby repetitive, sub-threshold stimuli induce episodes until they begin to occur spontaneously may be a feature of both epilepsy and BP (Amann and Grunze, 2005). This kindling phenomenon is thought to leave lasting physiological changes in the brain that predispose it to be more sensitive to further stressors. Further, AEDs that are used to treat both epilepsy and BP such as carbamazepine (Post et al., 1982), valproate (Loscher et al., 1989), and lamotrigine (O’Donnell and Miller, 1991) have been shown to exhibit anti-kindling properties.

Mood disorders are frequently comorbid with epilepsy (Mula et al., 2008). Earlier findings suggested that BP was rarely diagnosed in epilepsy patients. However, a large population-based survey in the United States found evidence of BP symptoms in 12.2% of epilepsy patients, and nearly half of these (6%) received a research-diagnosis of BP from a physician. This rate was higher than comorbidities of other medical disorders within the epileptic population (Ettinger et al., 2005). This comorbidity is puzzling given that epilepsy patients are treated with AEDs, which are potent mood stabilizers (Mula et al., 2008). Additionally, an affective syndrome, known as interictal dysphoric disorder (IDD), occurs in patients with epilepsy anywhere from 0.1 to 4.3% (Robertson, 1992). In a study of 143 outpatients with epilepsy (Mula et al., 2008), 11.8% received a DSM-IV (Diagnostic and Statistical Manual of Mental Disorders, Fourth Edition) diagnosis of BP, but only 1.4% were considered “pure” BP, unrelated to IDD, seizures, or AED treatment. Although IDD symptoms are thought to be physiologically distinct from the neurological symptoms of epilepsy (Gibbs, 1951), it is unclear how the etiology of these bipolar-like symptoms relates to BP itself. Regardless, it may be reasonable to speculate that the remarkable similarities in symptoms indicate some common mechanisms in the pathophysiology, potentially through ion channelopathies.

Further epidemiological support for etiological overlap between these disorders comes from a population-based family study of the comorbidity and familiarity between epilepsy and psychosis (Clarke et al., 2012). Among patients with epilepsy, the authors found an increased risk for broadly defined psychosis (5.5-fold increase in risk), schizophrenia (8.5-fold increase), and bipolar disorder (6.3-fold increase). Additionally, having a parent with epilepsy conferred a twofold increase risk for psychosis (compared to individuals without parental history of epilepsy), and conversely, a parental history of psychosis increased risk for epilepsy by 2.7-fold.

One final intriguing parallel between BP and epilepsy is the overlap in their treatments. Remarkably, many AEDs, including carbamazepine, lamotrigine, and valproate are effective in the treatment of BP. In fact, valproate is considered a first-line treatment for acute mania as well as prophylaxis. And carbamazepine and lamotrigine are other leading alternative mood stabilizers, should first-line monotherapy with lithium or valproate fail. Lamotrigine, in particular, has been shown to be relatively more effective against bipolar depression (Sachs et al., 2000). Although the mechanisms of action for these drugs in treating BP may not necessarily be the same as those in treating epilepsy (Rogawski and Loscher, 2004), the similarities between the disorders coupled with overlapping treatment regimes strongly suggest for at least some common pathophysiology.

## DISCUSSION

Over the past few decades, countless effort and resources have been invested into researching the genetic contribution to bipolar disorder. While no genes have been conclusively shown to cause the disorder, each discovery has revealed a small part of the fascinatingly complex genetic architecture of BP. Evidence implicating various genes related to the structure and regulation of ion channels had peppered earlier linkage and association literature. This class of genes has garnered renewed support in the GWAS era, as both single SNP and pathways based analyses continue to implicate ion channel functioning. The course of illness of BP, specifically its episodic nature, fits well with the channelopathy paradigm. Thus, these ion channel genes, which profoundly impact the neuronal activity, are an interesting group as there is both statistical evidence as well as biological plausibility implicating their involvement. Much of the focus has been on the potential role of calcium ion channels in BP. Here, we review evidence that potassium ion channels may also contribute to the etiology and deserve further investigation.

The identification of genes that contribute to BP susceptibility is important for a number of reasons. Elucidating the genetic underpinnings of BP may reveal new treatment targets, facilitating rational drug design that could produce more effective therapeutics with fewer side effects (U.S. Department of Energy Genome Program, 2009). It could also facilitate the development of diagnostic testing via genetic screening. This may have clinical implications, particularly in the case of disorders with genetic heterogeneity, whereby the course of treatment may depend on the precise etiology of the disorder (Ashcroft, 2000).

## Conflict of Interest Statement

The authors declare that the research was conducted in the absence of any commercial or financial relationships that could be construed as a potential conflict of interest.
